# Globotriaosylsphingosine (lyso-Gb3) might not be a reliable marker for monitoring the long-term therapeutic outcomes of enzyme replacement therapy for late-onset Fabry patients with the Chinese hotspot mutation (IVS4+919G>A)

**DOI:** 10.1186/s13023-014-0111-y

**Published:** 2014-07-22

**Authors:** Hao-Chuan Liu, Hsiang-Yu Lin, Chia-Feng Yang, Hsuan-Chieh Liao, Ting-Rong Hsu, Chiao-Wei Lo, Fu-Pang Chang, Chun-Kai Huang, Yung-Hsiu Lu, Shuan-Pei Lin, Wen-Chung Yu, Dau-Ming Niu

**Affiliations:** 1 Department of Pediatrics, Taipei Veterans General Hospital, No. 201, Section 2, Shih-Pai Road, Taipei 112, Taiwan; 2 Taiwan Clinical Trial Consortium in Fabry Disease, Taipei, Taiwan; 3 Institute of Clinical Medicine, National Yang-Ming University, Taipei, Taiwan; 4 Department of Pediatrics, Mackay Memorial Hospital, Taipei, Taiwan; 5 Department of Medicine, Mackay Medical College, New Taipei City, Taiwan; 6 Mackay Junior College of Medicine, Nursing and Management, Taipei, Taiwan; 7 Neonatal Screening Center, Chinese Foundation of Health, Taipei, Taiwan; 8 Department of Pediatrics, Cathay General Hospital, Taipei, Taiwan; 9 Pathology and Laboratory Medicine Department, Taipei Veterans General Hospital, Taipei, Taiwan; 10 Division of Cardiology, Department of Medicine, Taipei Veterans General Hospital, No. 201, Section 2, Shih-Pai Road, Taipei 112, Taiwan; 11 National Yang-Ming University, School of Medicine, No. 201, Section 2, Shih-Pai Road, Taipei 112, Taiwan

**Keywords:** Biomarker, Fabry disease, Globotriaosylsphingosine, IVS4 + 919G > A mutation, Outcome

## Abstract

**Background:**

In Taiwan, DNA-based newborn screening showed a surprisingly high incidence (1/875 in males and 1/399 in females) of a cardiac Fabry mutation (IVS4 + 919G > A). However, the natural course, long-term treatment outcomes and suitable biomarkers for monitoring the therapeutic outcomes of these patients are largely unknown.

**Methods:**

Fabry disease (FD) patients who had received enzyme replacement therapy (ERT) for more than 1 year were enrolled in this study from December 2008 to April 2013. Periodic echocardiography and serum globotriaosylsphingosine (lyso-Gb3) analysis were carried out. Before and after ERT, left ventricular mass index (LVMI) and serum lyso-Gb3 level were compared and the correlation between the change of LVMI and the change of serum lyso-Gb3 were also analyzed.

**Results:**

Thirty-six patients, in four patient groups, were enrolled: (1) 16 males with IVS4 + 919G > A mutation; (2) 7 females with IVS4 + 919G > A mutation; (3) 2 males with classical mutations; and (4) 11 females with classical mutations. The follow-up period was 13–46 months. There were significant LVMI reductions after ERT in all four groups after excluding confounding factors. However, interestingly, serum lyso-Gb3 decreased significantly in the early period after ERT in all groups, but increased gradually after an average of 11.1 months after ERT in late-onset male and female Fabry groups, even when their LVMI still decreased or remained stable. Furthermore, there was no correlation between the change of serum lyso-Gb3 and the change of LVMI in both classical and IVS4 + 919G > A FD patients.

**Conclusion:**

Although lyso-Gb3 has a high diagnostic sensitivity in late-onset Fabry patients and has a good response to ERT during the early stages, it might not be a reliable marker for monitoring the long-term therapeutic outcomes of ERT for late-onset Fabry patients with the Chinese hotspot mutation (IVS4 + 919G > A).

## Introduction

Fabry disease (FD) is an X-linked lysosomal storage disorder that results from deficient α-galactosidase A (α-Gal A) activity. This leads to progressive accumulation of globotriaosylceramide (Gb3) and related glycosphingolipids in lysosomes of the heart, kidneys, skin and brain. The clinical manifestations in classical FD patients are angiokeratomas, corneal opacities, hypohidrosis, cardiomegaly, renal impairment, acroparesthesias, gastrointestinal abnormalities and cerebrovascular events [1]. The incidence of FD has been reported as 1 in 40,000-117,000 live births in general population [2],[3]. During the past decade, late-onset phenotypes of FD, primarily involving the heart, kidneys or cerebrovascular system, have been reported. Patients with the cardiac variant often lack the classical symptoms of FD and present with left ventricular hypertrophy (LVH), arrhythmias or hypertrophic cardiomyopathy in the fifth to eighth decades of life [4]-[8].

**Figure 1:**
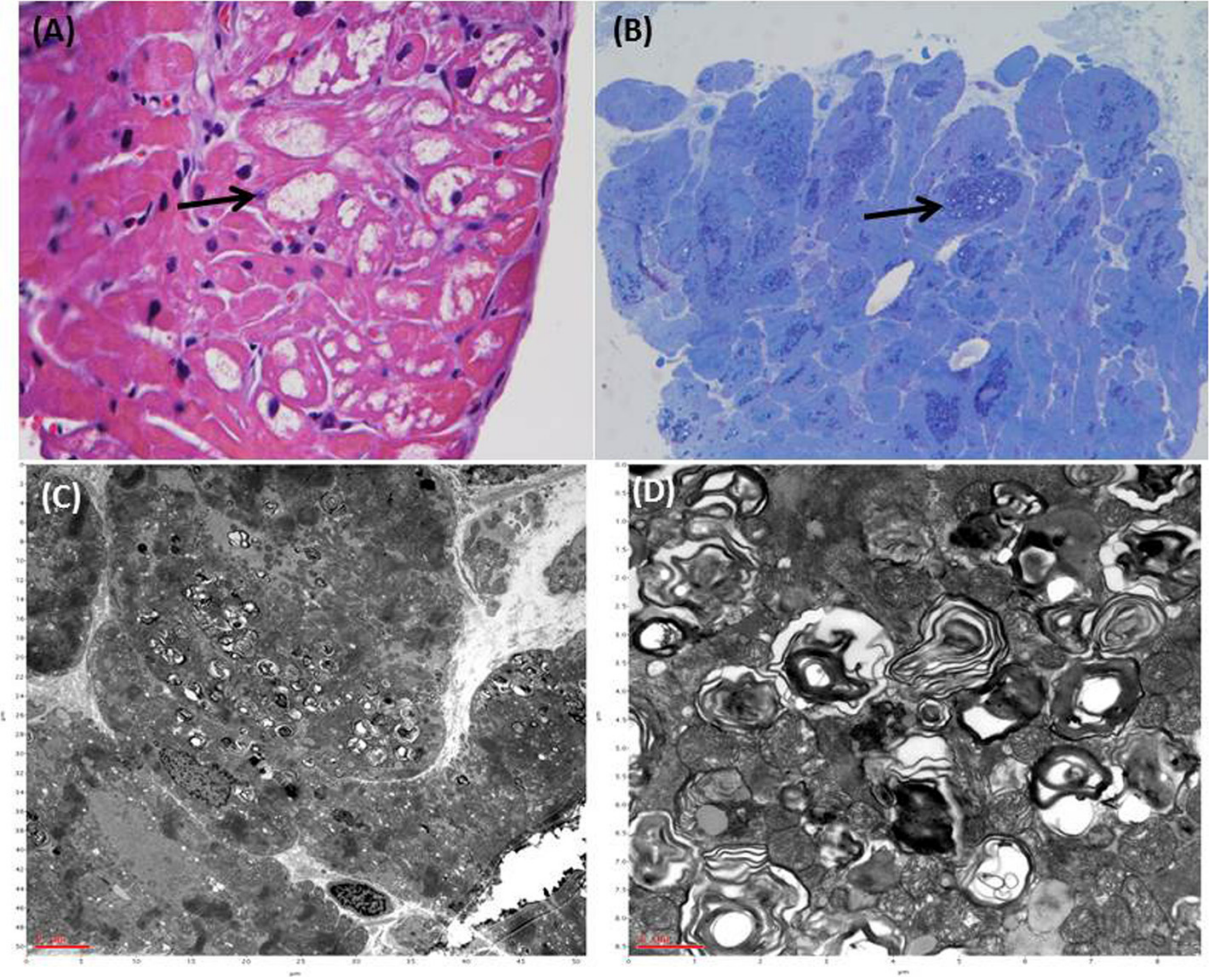
**Histological and electron microscopic results of cardiomyocytes of case 20, an IVS4 + 919G > A mutation male, obtained by endocardial muscle biopsy (A) Hematoxylin and eosin (H&E) staining showed a high degree of vacuolization (arrows) in cardiomyocytes due to lysosomal globotriaosylceramide (Gb3) accumulation. (B)** Toluidine blue stain revealed cytoplasmic granular inclusions (arrow) because of Gb3 accumulations. **(C) (D)** Electron microscopy revealed the characteristic Gb3 concentric lamellar myelin bodies in cardiomyocytes.

Our team first discovered the surprisingly high incidence of a cardiac *GLA* mutation, IVS4 + 919G > A, in Taiwan via newborn screening and further identified this mutation in idiopathic hypertrophic cardiomyopathy patients [9],[10]. Another DNA-based newborn screening program for this mutation in Taiwan revealed a higher incidence (1/875 in males and 1/399 in females) [11]. Patients who carried the IVS4 + 919G > A mutation and were older than 40 years had a higher prevalence of hypertrophic cardiomyopathy (72% of males and 35% of females) [12]. Endocardial biopsy of these patients with hypertrophic cardiomyopathy showed typical FD pathological change and significant Gb3 accumulation in the cardiomyocytes (Figure 1). However, the natural course, long-term treatment outcomes and suitable biomarkers for monitoring the therapeutic outcomes of these patients are largely unknown.

Our previous studies revealed that globotriaosylsphingosine (lyso-Gb3) has high diagnostic sensitivity and correlates with left ventricular mass (LVM), taking into account gender and age in our late-onset Fabry patients [13]. However, its use as a biomarker to monitor the therapeutic outcome of enzyme replacement therapy (ERT) has not been carefully evaluated in our late-onset Fabry patients. Several studies have indicated that serum lyso-Gb3 could be a biomarker for assessing ERT for FD. However, those studies were limited to classical Fabry patients and a short-term (<12 months) follow-up [14]-[16]. In our study, we evaluated the long-term response of lyso-Gb3 and left ventricular mass index (LVMI) to ERT in Fabry patients with classical or IVS4 + 919G > A mutations.

## Materials and methods

### Study population

This is a retrospective clinical study. From December 2008 to April 2013, all patients at our clinic with significant clinical manifestations of FD, and diagnosis confirmed by plasma α-Gal A enzyme assay and *GLA* gene mutation analysis were eligible for enrollment. Those who had available baseline echocardiography as well as serum lyso-Gb3 results and had received ERT for more than 12 months were enrolled in this study. Informed consents were obtained from patients or from patient’s parents if they were younger than 18 years. The study was approved by the medical ethics committee of Taipei Veterans General Hospital, Taiwan.

### Study protocol

All patients received ERT every 2 weeks with agalsidase beta (1 mg/kg) before 2010 and agalsidase alfa (0.2 mg/kg) since 2010, due to the shortage of agalsidase beta in Taiwan [17]. A comprehensive physical examination was carried out, echocardiography and serum lyso-Gb3 were evaluated prior to and during the follow-up. Patients were divided into four groups - classical male FD, classical female FD, IVS4 + 919 G > A male FD and IVS4 + 919 G > A female FD. Longitudinal results of LVMI and serum lyso-Gb3 were evaluated and compared between the baseline, first and latest follow-up after ERT.

### Echocardiography

Transthoracic echocardiographies were performed in accordance with American Society of Echocardiography (ACUSON Antares, Siemens, Germany; Sono 7500, Hewlett-Packard, USA). Standard two-dimensional M-Mode echocardiography was used from the left ventricular short axis view just below the mitral valve tip for diastolic interventricular septal thickness (IVSd), systolic and diastolic left ventricular internal diameter (LVIDs and LVIDd), and diastolic left ventricular posterior wall thickness (LVPWd). LVM was calculated according to the American Society of Echocardiography’s suggestion with the following equation: LVM(g) = 0.8 × (1.04 × [(LVIDd) + (IVSd) + (LVPWd)]^3^ − [LVIDs]^3^) + 0.6 [18]. LVM was normalized to height (meter) to 2.7 power (LVMI = LVM/ height^2.7^). Normal LVM was defined as 35 ± 8 g/m^2.7^ in men and 32 ± 8 g/m^2.7^ in women. LVH was defined as LVMI > 51 g/m^2.7^ in men and LVMI >48 g/m^2.7^ in women [19].

### Serum lyso-Gb3

The preparation and analysis of the samples was described in our previous publication [13]. Control samples were obtained from 31 healthy adults (16 males and 15 females). Serum lyso-Gb3 level <0.5 nM was defined normal. All the individuals in the control group had normal enzyme activity and no known GLA gene mutation.

Patient’s blood was used for lyso-Gb3 analysis. Plasma samples with 50 μL or calibration standards and internal standards were added into 96 wells plate. Lipid was extracted from plasma via chloroform, methanol and formic acid. Centrifuging was carried out several times during the extraction. Stepwise gradient elusion was done via a liquid chromatography–mass spectrometry system (Waters Alliance 2795XE HPLC, USA). Tandem mass spectrometry detection of lyso-Gb3 was performed on a triple quadruple mass spectrometer (Quattro Ultima, Waters, Milford, MA) with NeoLynx software version 4.1. Multiple reaction monitoring mode (MRM) was used for lyso-GB3 measurement.

In lyso-Gb3 analysis, we used lower (5 nM) and higher (100 nM) lyso-Gb3 spiked plasma as quality controls. Our laboratory’s coefficient of variation (CV) of inter-assay was 9.2% for the upper control and 11.0% for the lower control. As for intra-assay’s CV, it was 12.8% for the upper control and 9.9% for the lower control.

### Anti-agalsidase antibody

Blood was sent to SHIRE for anti-agalsidase antibodies examination. Specimens were screened and confirmed by a plate assay based upon ELISA technology, in which each patient’s sample was compared to the patient’s baseline. The ELISA which detected antibodies to Replagal is capable of detecting immunoglobulin isotype G (IgG) and E (IgE). Antibody positive cutpoint was absorbance ratio (time point/baseline) ≧2.0 and time point absorbance ≧ 0.040. In the absence of patient baseline, normal serum was used. The neutralizing antibody (Nab) positive cutpoint was >18% inhibition for Replagal. The lowest titer was 20.

### Statistical analysis

All statistical analyses were performed by Sigmastat version 3.5 (Dunas software LTD, Germany). The patients were divided into male and female groups and further divided into classical and IVS4 + 919G > A mutation groups. The continuous variables were compared using the paired student *t* test or Wilcoxon Signed Rank Test between the baseline, first and last follow-up data for LVMI and serum lyso-Gb3 level. Correlation analysis had been done via linear regression between the change of serum lyso-Gb3 and the change of LVMI from the baseline and last follow-up in all the included FD patients, classical FD patients, IVS4 + 919G > A FD patients, all males and all females regardless of the mutations. P < 0.05 was considered as statistically significant.

## Results

### Study population

Thirty-six patients (18 males and 18 females) were included in our study. Thirteen patients were classical FD (2 males and 11 females; c.1034 C > G: 1, c.394 G > A: 2, c.1081 G > T: 1, c.612 G > A: 6, c.1194delA: 3). Twenty-three patients had the IVS4 + 919G > A mutation (16 males and 7 females). The mean age was 57.8 years (range: 18.2-81.2 years). The follow-up time was 13–46 months (median: 20 months). Four of the FD patients (case 1, 14, 15, and 24) had received agalsidase beta before June 2009 and then changed to agalsidase alfa due to the shortage of agalsidase beta supply worldwide [17]. All of other patients only received agalsidase alfa treatment.

Eighteen patients (50%) had a history of hypertension; all of them received anti-hypertensive treatment and were normotensive (<140/90 mmHg) before receiving ERT. Six patients (16.7%) had a history of diabetes mellitus; five of them received oral hypoglycemic agents, one of them had regular insulin injections. Four patients (11.1%) had atrial fibrillation under medical control. Three patients (8.3%) had sick sinus syndrome and had received pacemaker implantation. One patient had lung adenocarcinoma and had received a complete course of chemotherapy. One patient stopped ERT by herself due to the improvement of LVH but re-started ERT owing to a remarkably elevated lyso-Gb3 level after ERT was discontinued. One patient had congestive heart failure which was under medical control.

### LVMI

The overall LVMI was reduced significantly (LVMI: 70.9 ± 28.7 g/m^2.7^ vs. 61.5 ± 28.6 g/m^2.7^, p < 0.001 ) after ERT while on follow-up. On sub-group analysis, LVMI was reduced significantly in both genders (male: 78.5 ± 31.2 g/m^2.7^ vs. 68. 3 ± 25.3 g/m^2.7^, p = 0.007; female: 63.3 ± 24.5 g/m^2.7^ vs. 54.6 ± 30.7 g/m^2.7^, p = 0.023). In the classical male group, both of the patients had a significantly reduced LVMI (57.2 ± 0.1 g/m^2.7^ vs. 46.8 ± 1.0 g/m^2.7^, p = 0.037). In the classical female group, interestingly, the initial analysis showed that the LVMI did not improve significantly (LVMI: 65.8 ± 30.1 g/m^2.7^ vs. 62.6 ± 36.9 g/m^2.7^, p = 0.533). However, three out of 9 female classical patients did not have LVH at the beginning of ERT (case 3, 4, and 6). One other classical female (case 13) had congestive heart failure before ERT and another classical female (case 12) had a non-ST segment elevation myocardial infarction (NSTEMI) during the course of ERT. The episode of NSTEMI was proved by the typical history of chest pain radiated to the back, T wave inversion over the inferior leads in electrocardiogram, and elevated serum troponin I which decreased gradually after management. Both of the conditions are risk factors for LVH. After excluding these 5 female patients, there was a significant improvement of LVMI in the classical female group. (LVMI: 66.7 ± 15.6 g/m^2.7^ vs. 55.47 g/m^2.7^, range: 53.7 ± 17.3 g/m^2.7^, p = 0.016) (Table 1).

**Table 1:** The LVMI and serum lyso-Gb3 changes after ERT in classical Fabry disease male and female patients

**Classical FD patients**	**Parameters, patient numbers**	**Baseline (Mean ± SD)**	**After ERT (Mean ± SD)**	**P value**
**Male**	LVMI (g/H2.7) N = 2	57.2 ± 0.1	46.8 ± 1.0	0.037*
	Compare between baseline and 1st Lyso-Gb3 (nM) N = 2	191.0 ± 17.9	76.1 ± 20.1	0.009*
	Compare between baseline and the last Lyso-Gb3 (nM) N = 2	191.0 ± 17.9	80.3 ± 15.3	0.010*
**Female**	LVMI (g/H2.7) N = 11	65.8 ± 30.1	62.6 ± 36.9	0.533
	LVMI (g/H2.7) N = 6**	66.7 ± 15.6	53.7 ± 17.3	0.016*
	Compare between baseline and 1st Lyso-Gb3 (nM) N = 11	16.3 ± 8.9	11.7 ± 6.2	0.005*
	Compare between baseline and the last Lyso-Gb3 (nM) N = 11	16.3 ± 8.9	11.7 ± 7.0	0.007*

*Asterisk represents statistically significant difference between the data of baseline and that after ERT (p < 0.05). ERT: enzyme replacement therapy; FD: Fabry disease; LVH: left ventricular hypertrophy; LVMI: left ventricular mass index; lyso-Gb3: globotriaosylsphingosine; SD: standard deviation.

**Case 3,4,6 were excluded in classical female FD’s LVMI comparison because these patient’s LVMIs were within normal limit at baseline. Case 12 and case 13 were excluded due to having risk factors for LVH.

### Serum lyso-Gb3 analysis

**Figure 2:**
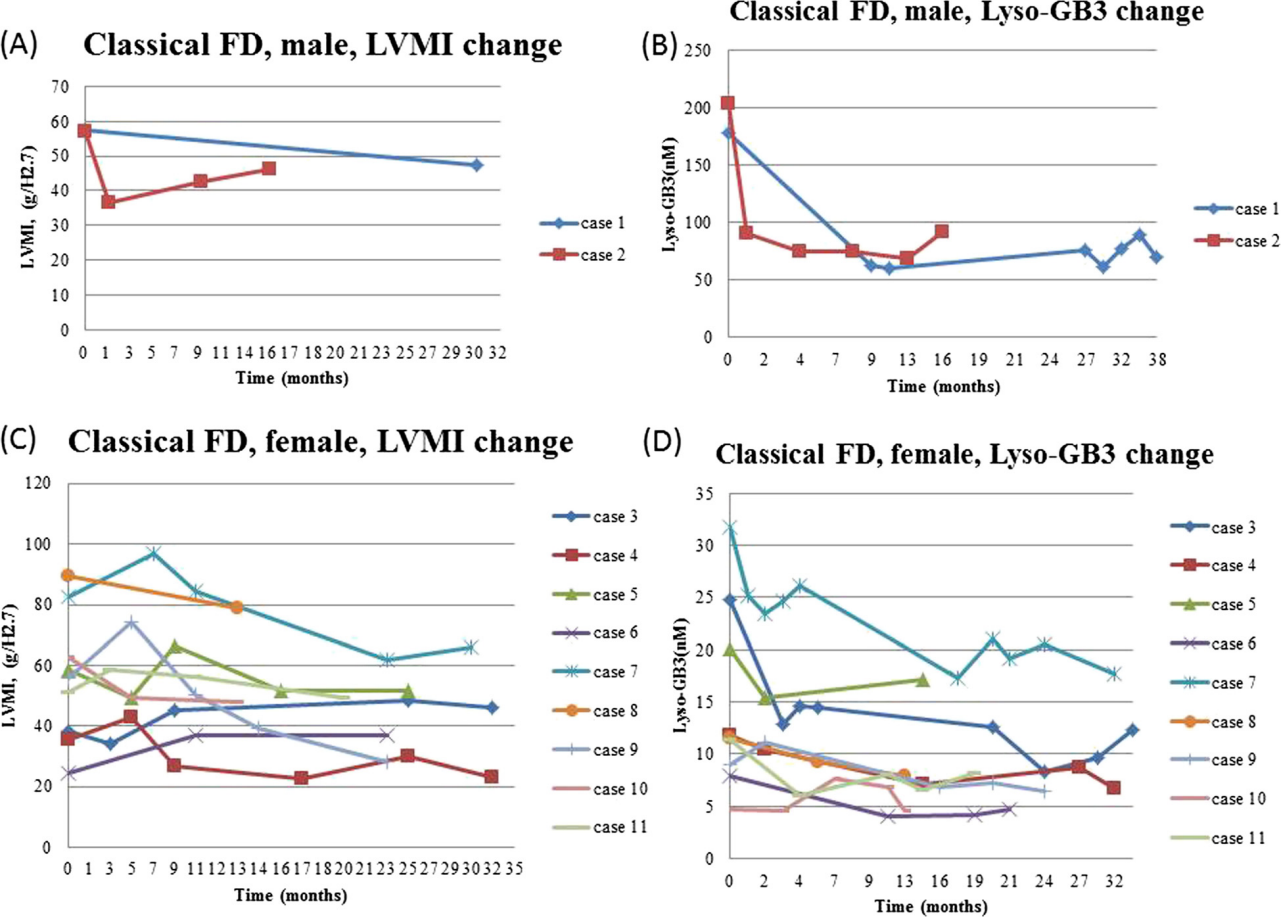
**The LVMI and serum lyso-Gb3 change after ERT in classical Fabry disease male (A) (B) and female (C) (D) patients.** Case 12 and case 13 were excluded due to having risk factors for left ventricular hypertrophy. FD: Fabry disease; LVMI: left ventricular mass index; lyso-Gb3: globotriaosylsphingosine.

**Figure 3:**
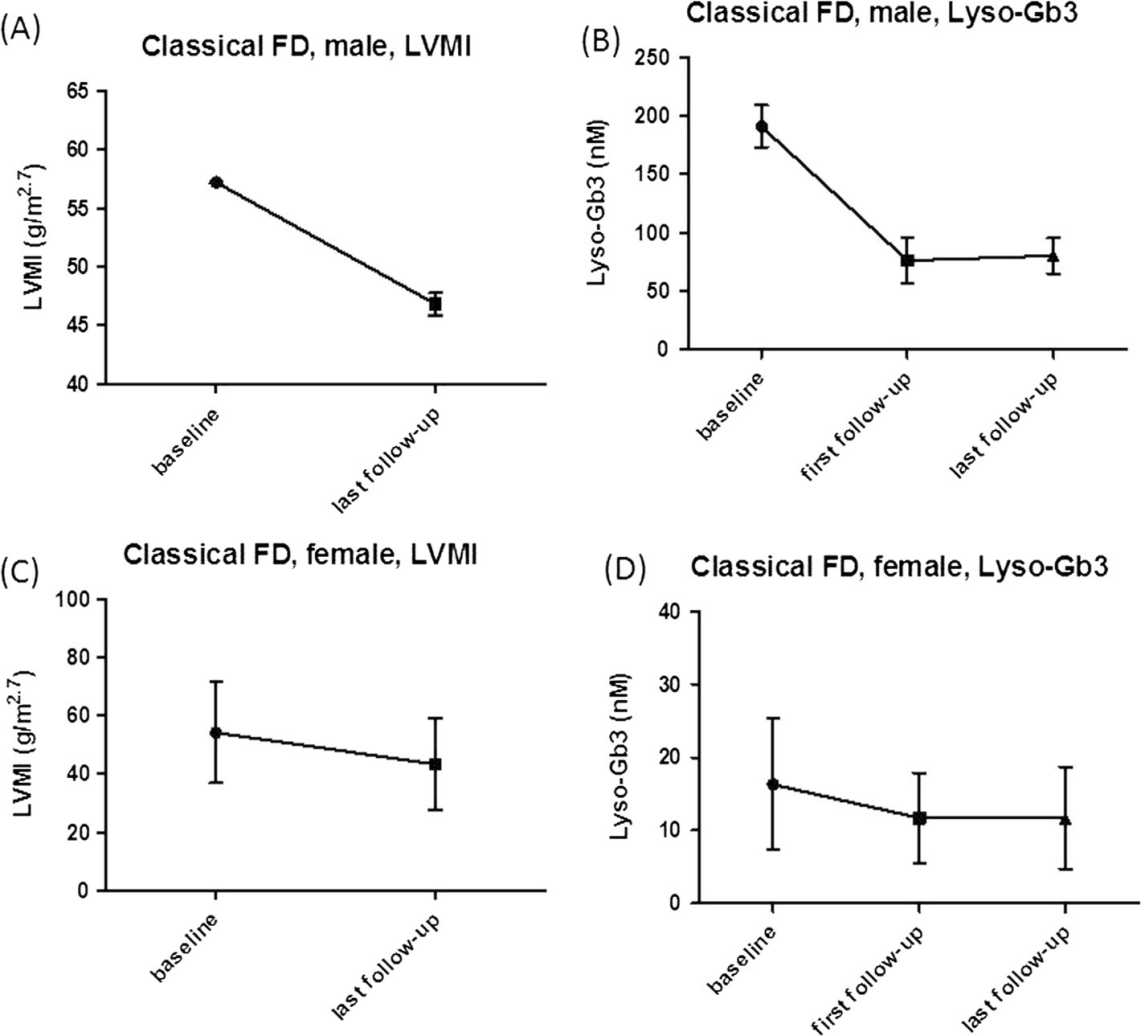
**In classical Fabry disease patients, the baseline and last follow-up LVMI (A)(C) as well as baseline, first and last follow-up serum lyso-Gb3 results (B)(D) were shown in mean ± SD.** Case 3, 4, 6, 12, 13 were excluded from classical females’ LVMI **(C)** due to their initial normal heart size or having risk factors for LVH. There was a significant decreased of LVMI between baseline and last follow-up in classical male **(A)** and female FD **(C)** patients. Significant decreased in serum lyso-Gb3 between baseline and first follow-up results as well as baseline and last follow-up results in classical male **(B)** or female **(D)** FD patients were found. FD: Fabry disease; LVMI: left ventricular mass index; lyso-Gb3: globotriaosylsphingosine; SD: standard deviation.

**Figure 4:**
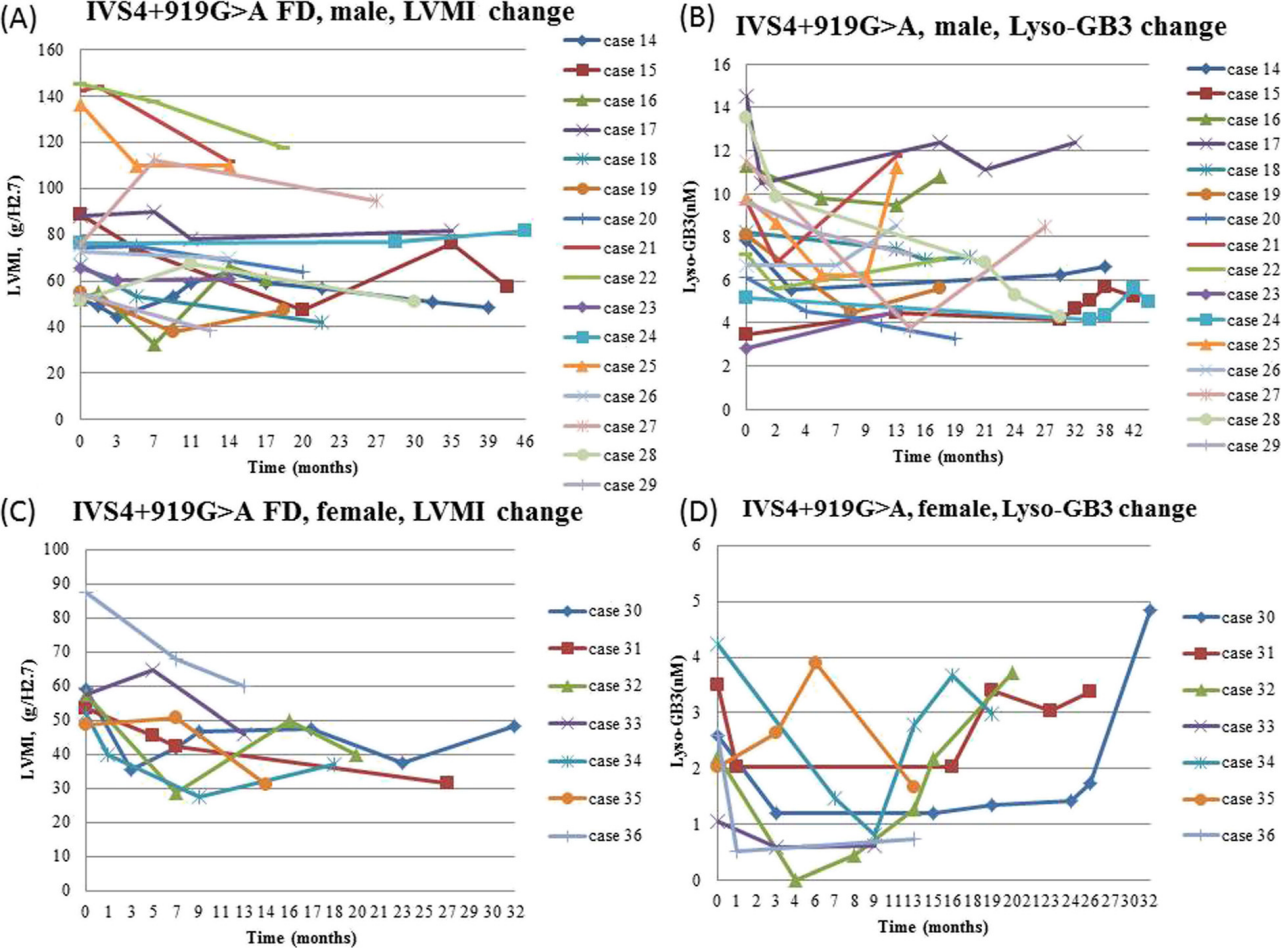
**The LVMI and serum lyso-Gb3 change after ERT in IVS4 + 919G > A Fabry disease male (A) (B) and female (C) (D) patients.** FD: Fabry disease; LVMI: left ventricular mass index; lyso-Gb3: globotriaosylsphingosine.

**Figure 5:**
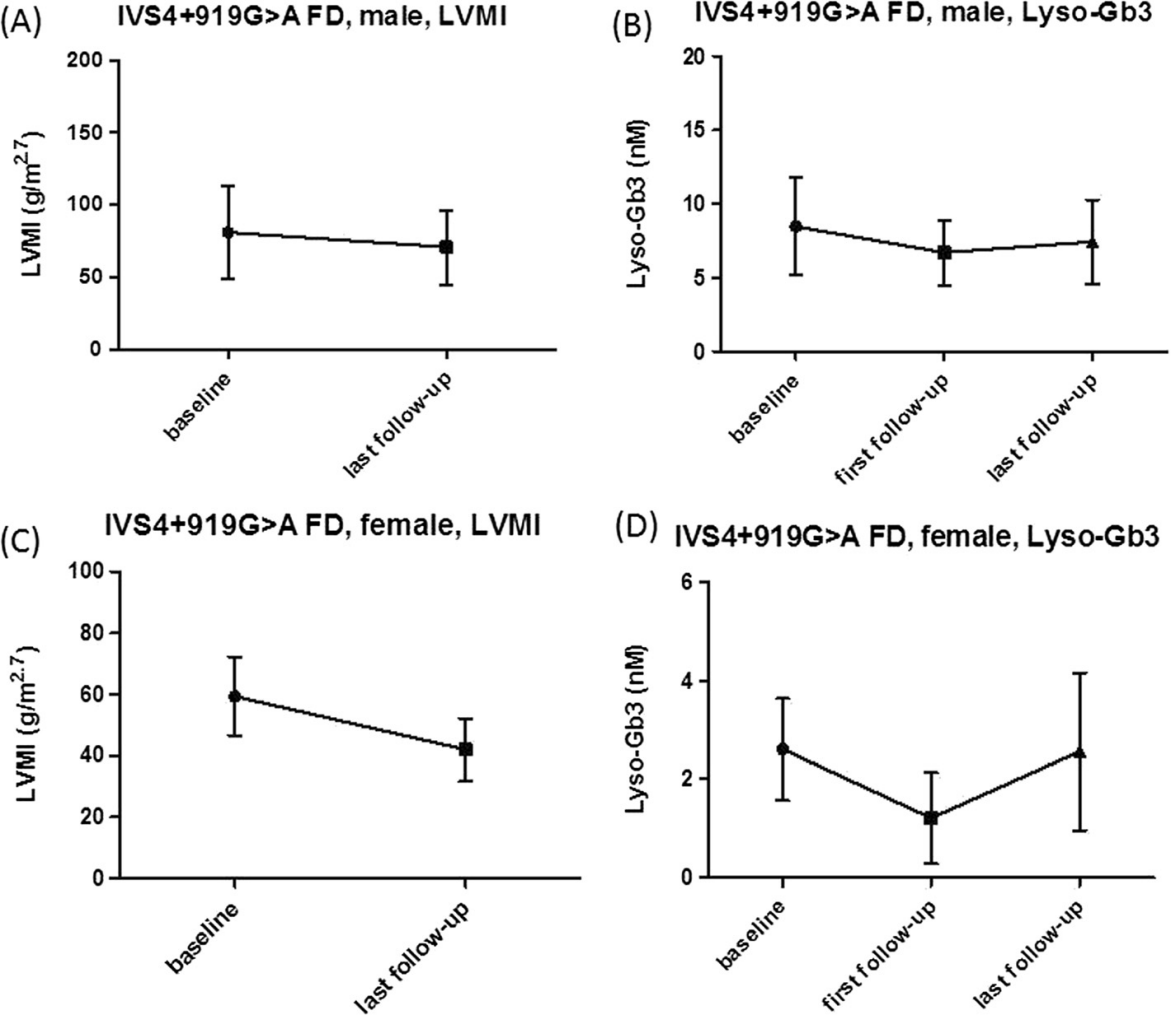
**In IVS4 + 919G > A Fabry disease patients, the baseline and last follow-up LVMI (A)(C) as well as baseline, first and last follow-up serum lyso-Gb3 results (B)(D) were shown in mean ± SD.** There were significant decreased of LVMI between baseline and last follow-up in IVS4 + 919G > A male **(A)** and female **(C)** FD patients. Significant decreased in serum lyso-Gb3 between baseline and first follow-up results were also found in IVS4 + 919G > A male **(B)** and female **(D)** patients. However, there were no statistical significance between baseline and last follow-up serum lyso-Gb3 results in IVS4 + 919G > A male **(B)** or female **(D)** FD patients. FD: Fabry disease; LVMI: left ventricular mass index; lyso-Gb3: globotriaosylsphingosine; SD: standard deviation.

After receiving ERT, the next serum lyso-Gb3 level was significantly decreased when compared with the baseline level in all groups (male FD: 8.91 nM, range: 2.81-203.68 nM vs. 6.74 nM, range: 3.79-90.31 nM, p = 0.002; female FD: 11.0 ± 9.7 nM vs. 8.0 ± 7.6 nM, p = 0.004; classical male FD: 191.0 ± 17.9 nM vs. 76.1 ± 20.1, p = 0.009 ; classical female FD: 16.3 ± 8.9 nM vs. 11.7 ± 6.2 nM, p = 0.005; IVS4 + 919 G > A male FD: 8.5 ± 3.3 nM vs. 6.7 ± 2.2 nM, p = 0.012; IVS4 + 919 G > A female FD: 2.6 ± 1.0 nM vs. 1.2 ± 0.9 nM, p = 0.018). (Figures 2, 3, 4 and 5)

However, in both males and females with the IVS4 + 919G > A mutation, the lyso-Gb3 level was found to be increasing slightly even when the LVMI of these patients was still improving or remaining stable after a longer follow-up time.(Figures 4 and 5) Therefore, we compared the baseline and the last serum lyso-Gb3 level in both male and female groups and found the significant differences in lyso-Gb3 levels disappeared between the baseline and the last follow-up (IVS4 + 919G > A male FD: 8.5 ± 3.3 nM vs. 7.4 ± 2.8 nM, p = 0.163; IVS4 + 919G > A female FD: 2.6 ± 1.0 nM vs. 2.6 ± 1.6 nM, p = 0.938) even when the significant differences in LVMIs still existed between the baseline and the last follow-up (IVS4 + 919G > A male FD: 81.1 ± 32.2 g/m^2.7^ vs. 71.0 ± 25.6 g/m^2.7^, p = 0.016; IVS4 + 919G > A female FD: 59.4 ± 12.8 g/m^2.7^ vs. 42.0 ± 10.2 g/m^2.7^, p < 0.001) (Table 2). We found that there was a trend in lyso-Gb3 levels during long-term ERT for most of our patients. In the beginning, there was a good response of lyso-Gb3 to ERT. The mean lowest serum lyso-Gb3 level was found around 11.1 months (median: 9 months) after ERT and then lyso-Gb3 level elevated gradually, even when the LVMI was still reducing or remaining stable. Therefore, at the last follow-up, compared with baseline, the significant differences in lyso-Gb3 levels had disappeared, but the significant differences in LVMI still existed.

**Table 2:** The LVMI and serum lyso-Gb3 change after ERT in IVS4 + 919G > A Fabry disease male and female patients

**Cardiac variant, IVS4 + 919G > A FD patients**	**Parameters, patient number**	**Baseline (Mean ± SD)**	**After ERT (Mean ± SD)**	**P value**
**Male**	LVMI (g/H^2.7^) N = 16	81.1 ± 32.2	71.0 ± 25.6	0.016*
	Compare between baseline and 1^st^ Lyso-Gb3 (nM) N = 16	8.5 ± 3.3	6.7 ± 2.2	0.012*
	Compare between baseline and the last Lyso-Gb3 (nM) N = 16	8.5 ± 3.3	7.4 ± 2.8	0.163
**Female**	LVMI (g/H^2.7^) N = 7	59.4 ± 12.8	42.0 ± 10.2	<0.001*
	Compare between baseline and 1^st^ Lyso-Gb3 (nM) N = 7	2.6 ± 1.0	1.2 ± 0.9	0.018*
	Compare between baseline and the last Lyso-Gb3 (nM) N = 7	2.6 ± 1.0	2.6 ± 1.6	0.938

*Asterisk represents statistically significant difference between the data of baseline and that after ERT (p < 0.05). ERT: enzyme replacement therapy; FD: Fabry disease; LVMI: left ventricular mass index; lyso-Gb3: globotriaosylsphingosine; SD: standard deviation.

Similarly, we also found the classical patients had a very good response in lyso-Gb3 levels during the early period of ERT. The mean lowest serum lyso-Gb3 level was found around 13.3 months after ERT. Thereafter, the lyso-Gb3 level started to increase gradually, but the increase was not as significant as in our cardiac variant patients.

### Anti-agalsidase alfa antibody analysis

Twenty-three patients received serum anti-agalsidase alfa analysis. Only one classical male (case 1) and one classical female (case 12) had positive anti-agalsidase alfa IgG, but IgE and NAb were all negative in both of the patients. None of the IVS4 + 919G > A patients had anti-agalsidase alfa antibody.

### Correlation analysis between serum lyso-Gb3 and LVMI

There were no significant correlation between the change of LVMI and the change of serum lyso-Gb3 from baseline to the last follow-up in all the included FD patients (r = 0.002, p = 0.912), classical FD patients (r = 0.191, p = 0.531), IVS4 + 919G > A FD patients (r = 0.364, p = 0.088), all males (r = 0.024, p = 0.926) and all females (r = 0.167, p = 0.51) regardless of the mutations.

## Discussion

From our Fabry newborn screening program, we found a surprisingly high prevalence of a *GLA* mutation, IVS4 + 919G > A, in our population (1/875 in males and 1/399 in females) [11]. In addition to Taiwan, this mutation was also found in Mainland China, Singapore (Han population), and Malaysia (Han population) (unpublished data). Therefore, we believe the IVS4 + 919G > A mutation may be the most common pathogenic mutation of FD in the world. It is very important to understand the natural course of the disease caused by this mutation, as well as treatment outcomes and suitable biomarkers for monitoring ERT.

Lyso-Gb3, a deacylated Gb3, has been shown to have a proliferative effect on smooth muscle cells. Elevated lyso-GB3 levels have been found in classical and late-onset FD patients [16],[20]-[23]. ERT can dramatically decrease serum lyso-Gb3 levels. Rombach et al. found a relationship between serum lyso-Gb3 level and the severity of classical FD [20],[21]. In our previous study, we found LVMI and lyso-Gb3 levels in patients with IVS4 + 919G > A mutation had a good response to ERT during the early treatment period [16]. However, the responses to a longer-term treatment have not been previously analyzed. In this study, we analyzed the long-term responses of LVMI and lyso-Gb3 to ERT in our patients with IVS4 + 919G > A mutation. To our surprise, the significant difference in lyso-Gb3 levels disappeared gradually between baseline and follow-up after long-term ERT, even when the LVMI of these patients were still improving or remaining stable. The reasons for the elevation of lyso-Gb3 levels remain unknown. We suspect that there are some tissues, which might not cause significant manifestations of FD, that respond poorly to ERT and the accumulation of lyso-Gb3 was still in progress. Furthermore, lyso-Gb3 is only very marginally elevated in our IVS4 + 919G > A patients. Due to the very low range, there might be an unacceptable degree of assay variation, which would render this marker unsuitable for follow-up.

For classical patients, we did not find as significant an elevation of lyso-Gb3 levels as in patients with IVS4 + 919G > A mutation during long-term follow-up. Because the lyso-Gb3 levels of classical patients were much higher than those with IVS4 + 919G > A mutations, we suspect that the amount of lyso-Gb3 accumulation in the tissues responding poorly to ERT was too small to affect the serum lyso-Gb3 levels.

When we further analyzed the correlation between the change of LVMI and the change of serum lyso-Gb3 in different FD patient groups, we found no significant correlations between the changes of these two parameters while longer follow-up. It means a mark decreased serum lyso-Gb3 does not indicate a greater improvement of cardiac size. Although Rombach et al. [21] had reported a good correlation between plasma lyso-Gb3 and LVM in female classical FD patients before ERT, serum lyso-Gb3 cannot be used as a long-term monitor for the change of LVMI in both classical and cardiac variant FD patients after ERT.

Our study reveals that serum lyso-Gb3 could be a good biomarker for the response to ERT in the early stages of treatment for patients with the IVS4 + 919G > A mutation. However, serum lyso-Gb3 loses its reliability for monitoring the therapeutic outcome to ERT in these cardiac variant patients.

There were several limitations in this study. First, the number of patients in each group was not large enough, especially for the classical patients, to make a solid conclusion. Although the echocardiographic examination was performed via a standard protocol, employing a single echocardiographic machine and only two skilled cardiologists, the variability of LVMI measurements still needs to be considered. Furthermore, the serum lyso-Gb3 levels of the patients with IVS4 + 919G > A mutation were very low. Although we did our best to maintain experimental stability and consistency and kept our intra-assay and inter-assay CV values within the acceptable range, the CV of our laboratory still had an influence on the interpretation of the results.

## Conclusion

Even lyso-Gb3 has a high diagnostic sensitivity in FD patients and has a good response to ERT in early stages, it might not be a reliable follow-up marker for monitoring the long-term therapeutic result of ERT in late-onset FD patients with the Chinese hotspot mutation, IVS4 + 919G > A.

## Abbreviations

α-Gal A: α-galactosidase A

CV: Coefficient of variation

ERT: Enzyme replacement therapy

FD: Fabry disease

Gb3: Globotriaosylceramide

IgG: Immunoglobulin isotype G

IgE: Immunoglobulin isotype E

IVSd: Diastolic interventricular septal thickness

LVH: Left ventricular hypertrophy

LVIDd: Diastolic left ventricular internal diameter

LVIDs: Systolic left ventricular internal diameter

LVM: Left ventricular mass

LVMI: Left ventricular mass index

LVPWd: Diastolic left ventricular posterior wall thickness

Lyso-Gb3: Globotriaosylsphingosine

MRM: Multiple reaction monitoring mode

Nab: Neutralizing antibody

NSTEMI: Non-ST segment elevation myocardial infarction

## Competing interests

The authors declare that they have no competing interests.

## Authors’ contributions

HCL and HYL performed acquisition, statistical analysis and interpretation of data, and drafting of the manuscript. WCY and DMN participated in design of the study, interpretation of the data and helped to draft the manuscript. FPC performed the pathological interpretation of cadiomyocytes. HCL, CKH, and YHL performed biochemical analyses and revised the manuscript. SPL, CFY, TRH, and CWL were responsible for patient screening. All authors read and accepted the manuscript.
